# Supplementary material for: Modeling glioblastoma heterogeneity as a dynamic network of cell states
Source: Mol Syst Biol. 2021 Sep 16;17(9):e10105. doi: 10.15252/msb.202010105 (PMC8444284; doi:10.15252/msb.202010105)
Supplement: Supplementary file 6 — Source Data for Figure 5 [file MSB-17-e10105-s004.zip › Figure5A_sourcedata/GSEA_3017/nosigdb_stateE.GseaPreranked.1622795031093/OLSEN_SCPLIKE.html]

Details for gene set OLSEN\_SCPLIKE[GSEA]

|  || Dataset | state63017 |
| Phenotype | NoPhenotypeAvailable |
| Upregulated in class | na\_pos |
| GeneSet | OLSEN\_SCPLIKE |
| Enrichment Score (ES) | 0.71411157 |
| Normalized Enrichment Score (NES) | 3.7314603 |
| Nominal p-value | 0.0 |
| FDR q-value | 0.0 |
| FWER p-Value | 0.0 |
Table: GSEA Results Summary

  

Fig 1: Enrichment plot: OLSEN\_SCPLIKE      
 Profile of the Running ES Score & Positions of GeneSet Members on the Rank Ordered List

  

| PROBE | GENE SYMBOL | GENE\_TITLE | RANK IN GENE LIST | RANK METRIC SCORE | RUNNING ES | CORE ENRICHMENT || 1 | PMP22 |  |  | 0 | 1.393 | 0.1039 | Yes |
| 2 | IGFBP5 |  |  | 4 | 1.015 | 0.1763 | Yes |
| 3 | ANXA1 |  |  | 12 | 0.826 | 0.2304 | Yes |
| 4 | CRYAB |  |  | 16 | 0.788 | 0.2859 | Yes |
| 5 | ANXA2 |  |  | 18 | 0.733 | 0.3394 | Yes |
| 6 | CNN3 |  |  | 24 | 0.702 | 0.3864 | Yes |
| 7 | LGALS3 |  |  | 25 | 0.697 | 0.4383 | Yes |
| 8 | IFITM3 |  |  | 26 | 0.682 | 0.4892 | Yes |
| 9 | PMEPA1 |  |  | 44 | 0.544 | 0.5114 | Yes |
| 10 | IFITM2 |  |  | 46 | 0.536 | 0.5502 | Yes |
| 11 | S100A10 |  |  | 51 | 0.527 | 0.5852 | Yes |
| 12 | CST3 |  |  | 86 | 0.425 | 0.5800 | Yes |
| 13 | CD59 |  |  | 89 | 0.418 | 0.6090 | Yes |
| 14 | GPM6B |  |  | 105 | 0.391 | 0.6219 | Yes |
| 15 | TIMP3 |  |  | 106 | 0.391 | 0.6510 | Yes |
| 16 | CD99 |  |  | 107 | 0.389 | 0.6800 | Yes |
| 17 | LGALS1 |  |  | 113 | 0.368 | 0.7021 | Yes |
| 18 | VMP1 |  |  | 127 | 0.351 | 0.7141 | Yes |
| 19 | S100A6 |  |  | 176 | 0.312 | 0.6854 | No |
| 20 | GAS7 |  |  | 180 | 0.310 | 0.7052 | No |
| 21 | SPTBN1 |  |  | 194 | 0.300 | 0.7135 | No |
| 22 | ITGB8 |  |  | 239 | 0.268 | 0.6858 | No |
| 23 | QKI |  |  | 241 | 0.267 | 0.7046 | No |
| 24 | ZFP36L1 |  |  | 287 | -0.255 | 0.6749 | No |
| 25 | BTG1 |  |  | 772 | -0.520 | 0.1887 | No |
Table: GSEA details [plain text format]

  

Fig 2: OLSEN\_SCPLIKE: Random ES distribution      
 Gene set null distribution of ES for **OLSEN\_SCPLIKE**
